# Sleeping Sickness

**Published:** 1908-01-04

**Authors:** George C. Low

**Affiliations:** Lecturer in Tropical Diseases to the College, etc.


### Hospital Clinics.

### SLEEPING SICKNESS.

By GEORGE C. LOW, M.A., M.B., Lecturer in Tropical Diseases to the College, etc.

A Lecture delivered at the Post-Graduate College, West London Hospital, November 5, 1907.

### Definition.

Sleeping sickness may now be defined as the terminal phase of human trypanosomiasis, a disease endemic in different parts of equatorial Africa: characterised, in the initial stages, by irregular remitting fever, increased pulse-rate and respiration, œdemas, and irregular erythematous rashes; in the later stages (sleeping-sickness) by gradually increasing lethargy, mental and physical degeneration, progressive emaciation, tremors, and other nervous phenomena, and death.

### History.

The first recorded account of sleeping sickness was published by Winterbottom in 1803. Later, Clarke gave a short description of the disease, and from that time, 1840, up till 1900, many other observers have added to our knowledge. At that later date fresh and increased attention was directed to it on account of the study of two cases in Charing-Cross Hospital by Sir Patrick Manson, Dr. Mott, and others, and here, for the first time, the pathological anatomy was thoroughly and accurately described. In 1902, owing to the enormous and rapid spread of the disease in Uganda, the Foreign Office and the Royal Society sent out a Special Commission, and the work done by that and subsequent bodies has completely elucidated the etiology of the disease. Before this, on December 18, 1901, Dutton had discovered trypanosomes in the blood of a European, a patient of Forde's in the Gambia Colony, and had followed this up by giving an accurate account of the clinical features of the case, but neither he, Todd, Brumpt, nor Baker, who also found trypanosomes in human cases later, associated the disease with sleeping sickness; the explanation of this we shall see later. At the beginning of 1903 Castellani, one of the members of the Uganda Commission, when centrifuging the spinal fluid for bacteria, found a trypanosome there and verified this in several other cases. At this important juncture Colonel Bruce arrived, and on hearing of Castellani's discovery at once confirmed and amplified it. Together with Nabarro and others he demonstrated that trypanosomes can be found in every case of sleeping sickness, not only in the cerebro-spinal fluid, but also in the blood, that the parasite is the same as that discovered by Dutton on the Gambia, that human trypanosomiasis is the first stage of sleeping sickness, and that a tsetse fly, *Glossina palpalis*, is implicated in the spread of the disease. The reason why Dutton and others did not detect that the two diseases are one is simple; the symptoms of sleeping sickness only appear in most cases years after the initial infection, and, as Bruce believed, when the parasites leave the blood and enter the cerebro-spinal fluid. Since that date ample confirmation of all those points has been obtained and further information acquired, and just 100 years after the first recorded account of sleeping sickness was published, science at last demonstrated the cause.

### Etiology.

Before the discovery of the trypanosome many hypotheses had been brought forward to explain the genesis of sleeping sickness. It was supposed by some to be a food intoxication, by others to be due to animal parasites, and yet by others to be bacterial in its origin. The latter only need be mentioned here. A Portuguese Commission in 1901 found a diplo-streptococcus in many cases of the disease, and concluded that it was the cause. In the following year Castellani, in Uganda, demonstrated the same organism, more especially, however, in the later stages of the disease. We now know that those germs play only a subsidiary part. They generally appear towards the termination of the malady, and are much on a par with the pneumococcus, bacillus coli, etc., which may act in many cases as the immediate producers of death.

### Geographical Distribution.

The endemic area of human trypanosomiasis is limited to parts of equatorial Africa, and no case has been known except among those who have been resident within this area. On the West Coast of Africa the disease is found from Senegal in the north to San Paolo de Loanda, or even beyond, in the south, the Congo State and Uganda being also affected. The distribution within those bounds varies, however, considerably; it is not, for example, common in the Gambia, the Gold Coast, the Niger protectorates, and Lagos, whereas on the Congo and Uganda it has simply decimated the population, and is spreading quickly into regions previously exempt. As Colonel Bruce has shown, in Uganda it keeps to the lake shores, the haunts of a tsetse fly, known by the name of *glossina palpalis*, and its distribution in other parts of Africa must depend on the presence or absence of this fly, or of others of the same genus. It is not known yet in British East Africa east of the Mau Escarpment, nor in the coastal parts of German East Africa, Rhodesia, British Central Africa, and the southern parts of Africa. The Sudan and Egypt are still also free, but the hinterlands of most of the West Coast colonies have recently furnished cases.

The trypanosomes are protozoal parasites of the division mastigophora or flagellata. They are elongated, unicellular organisms, measuring about 20 to 25 $\mu$. in length by about 2.5 $\mu$. in breadth. Anteriorly they are furnished with a whip-like process, the flagellum, which is continued along the body and ends near the posterior extremity. In its course it is thickened and thrown into folds forming what is known as the undulating membrane. About the centre is a compact mass of chromatin, the nucleus, and almost at the posterior end is a deeply-staining spot, the centrasome, a clear unstaining area just in front of this being termed the vacuole. Amidst the body protoplasm other granular spots may be detected after suitable staining, but whether those are evidences of degeneration or not is not quite clear. To study the trypanosomes either fresh blood specimens may be examined to see movements and other features, or stained films to show the various anatomical features described above. For staining one of the modifications of Romanowsky's chromatin staining method must be employed, and the most simple and practical of those is Leishman's modification. In addition to man, many other animals suffer from diseases due to trypanosomes, and a comparative study of those forms is essential to the proper understanding of the human variety. Of those the commoner varieties are (1) "*Surra*," a disease of horses and mules, found in India, Burma, the Philippines, and Mauritius; (2) "*Nagana*," or *tsetse fly disease*, a malady attacking horses and oxen in various parts of Africa; (3) *Mal de Cadêras*, a disease of horses, found in Central South America; and (4) *Dourine*, a disease of brood mares and stallions, found in Algiers, India, the South of Spain, etc. Some authorities believe that the first three diseases are one and the same, and due to the same parasite, while others are equally emphatic that they are different and that the parasites are specifically distinct. Morphologically the three trypanosomes certainly closely resemble each other, and they can only be separated by serial inoculations into different animals. The names of the most important trypanosomes are as follows:—

*Trypanosoma gambiense*=the parasite of sleeping sickness.

*Trypanosoma evansi*=the parasite of Surra.

*Trypanosoma bruceii*=the parasite of tsetse-fly disease or Nagana.

*Trypanosoma equini*=the parasite of mal de Cadêras.

*Trypanosoma equiperdum*=the parasite of Dourine. Others are also known. The carriers of these organisms from one host to another, with the exception of dourine, which is spread by coitus, are biting flies; in Africa of the genus *glossina* (tsetse flies), in India, as far as we know, of two genera, *Stormoxys* and *Tabanus*.

The part played by tsetse flies is interesting historically and is worth narrating. In the old days residents in parts of South Africa, together with explorers and sportsmen, were aware of the fact that if they took cattle or horses through certain belts of country the latter got bitten by tsetse flies and died as a result, and so the name arose of tsetse fly belts. It was supposed the flies were poisonous, but the nature of the toxin injected was quite unknown until Colonel Bruce, in Zululand, solved the riddle by discovering that the fly of itself is not poisonous, but that it acts merely as the carrier of a parasite, a trypanosome, from the sick to the healthy, the sick or infected animals in this instance being the wild game. Apparently little inconvenienced by those parasites themselves, the wild animals nevertheless infect the flies, and they, in their turn, the susceptible animals, oxen and horses, passing through the belts. By carefully thought-out and very accurately conducted experiments, Colonel Bruce was able to show that other biting flies do not spread the infection from animal to animal, and the tsetse flies only if they bite the fresh animal within a short period after feeding on the infected one. This indicates that the fly acts as a purely mechanical carrier, and that no development takes place in its interior, as in the case of malaria in the mosquito; in other words, that it is not a host in the true sense of the term.

As soon as trypanosomes were discovered in sleeping sickness Colonel Bruce concluded at once from his previous work on Nagana that a tsetse fly would prove to be the transmitter, and in the affected regions such a fly (*glossina palpalis*) was quickly discovered. Other experiments led to the conclusion that this tsetse is the sole carrier of the human trypanosome, other biting flies being harmless: the definite geographical range of the two was found to coincide perfectly in every way. In the original nagana experiments little was known of the different species of tsetse flies, the term tsetse fly covering them all, but now Mr. Austen believes that two distinct species were used, *glossina morsitans* and *glossina pallidipes*, so we may take it that both of those may spread the *T. brucei*. This being so, can those two species or others also spread the *T. gambiense*, or, conversely, can *glossina palpalis* spread nagana? These points are not yet settled, but in view of the rapid spread of sleeping sickness a decision is urgently required, and for the present it is best to view all tsetse flies with suspicion as possible carriers of the human trypanosome.

The numbers of trypanosomes found in the peripheral blood varies very greatly. In human cases they are generally very scanty; in nagana and surra they are more numerous, tending to become less so, however, when the disease becomes chronic. In experimental animals, rats and mice, an infection with *T. brucei* quickly ends fatally (about ten days for rats), and then the parasites are almost as numerous as the red blood corpuscles. Even though no parasites are found in any given individual or animal which has once had them, the blood may still be infectious when inoculated into suitable experimental animals, showing that the infection is still present and still virulent. In human cases the *T. gambiense*, in addition to occurring in the blood, is also present in the lymphatic system, and examination of the fluid drawn from a lymphatic gland is a more certain method of diagnosis than blood examination.

### Symptoms and Clinical Features.

For convenience in classification we may divide trypanosomiasis in the human subject as follows:—I. The phase of trypanosomiasis fever; II. The phase of sleeping sickness; the latter may run an acute or chronic course, being still further sub-divided into three stages: (1) The stage of indefinite symptoms, vague pains, feelings of indisposition, increasing languor, slight degrees of drowsiness, and raised temperature; (2) the stage of increasing drowsiness, more marked lethargy, tremors, and other definite phenomena; (3) the terminal stage of emaciation, intense weakness, aggravation of the former symptoms, gradually deepening coma, sub-normal temperature, and death. Those different phases merge insensibly into each other, much in the same way as do the three stages of syphilis, and this, of course, must not be forgotten in the study of individual cases.

After the inoculation of the trypanosome into the system a series of well-defined clinical symptoms sooner or later appears, the incubation period, probably a short one, being in many instances very difficult to determine. In the first case in a European described by Dutton, and subsequently in others described by Manson, the following cardinal points were noted: (1) An irregular remitting or intermitting temperature; (2) an increase in the pulse-rate from the normal 70 to 120 or more beats per minute; (3) an increase in the respiration-rate to 20 or 30; (4) irregular patches of a congested or erythematous character, often somewhat circinate in contour, the colour returning after pressure, distributed on different parts of the body; (5) œdema of the legs or face, or both, varying in degree from a scarcely noticeable swelling to a well marked puffiness. In natives the erythematous condition is absent or, at least, not detectable. Stephens, in summing up the prognosis of this phase of the disease, states: "This is grave in the European, though apparently recovery may occur. The disease may terminate in sleeping sickness or by intercurrent disease." In determining those points the histories of some of the recorded European cases are interesting. Dutton's first case died without exhibiting any signs of sleeping sickness; a case seen by Manson in England and also reported by Broden on the Congo is still alive, and apparently perfectly healthy six years or so after trypanosomes were seen in her blood; another case of Manson's died two years after the trypanosome infection, and in her last days showed undoubted signs of sleeping sickness, verified satisfactorily at the autopsy; another case of Bruce's and Bradford's also ended in sleeping sickness. So it may be looked upon as certain that when definite signs of sleeping sickness, such as drowsiness and lethargy appear, the individual is doomed.

Sleeping sickness begins very insidiously, often months or even years after the original trypanosome infection. In addition to the languor and drowsiness marked changes occur in the disposition of the patient. Natives are very quick at noticing these points, and they will bring patients to the doctor with the diagnosis correct time after time, though to the ordinary observer there may be little to be seen. Quite early there may be a drooping of the upper eyelids, slight puffiness of the face, and a gradually increasing change in the facial aspect; a previously happy and intelligent-looking negro becoming sad, apathetic and morose. Next appears a disinclination to work, with a desire to sit about and rest more than usual, and at this time headaches and other transient pains, especially in the upper part of the chest, may be complained of. A tendency to fall asleep at unusual times, even in spite of strong efforts to keep awake, and a desire to lie and doze in the sun may also be noticed.

The appearance of the face is dull, heavy, and stupid. Questions are answered, but slowly, the speech, when it does come, often being mumbled, slow, and thick. The gait is peculiar; the patient, instead of lifting his feet in the ordinary manner, pushes them along the floor as he advances. Even at this early stage a fine fibrillary tremor may be detected in the tongue and, in some instances, also in the muscles of the hands. Glandular enlargements may be so prominent as to be evident without palpitation; but in other cases they are smaller and less noticeable.

The patient soon gets worse, he stays in bed more, muscular weakness and wasting become pronounced, tremors are more marked (tongue, muscles of arms, hands, and even legs), and the drowsiness and lethargy increase. Later the skin may lose its lustre, becoming dry and coarse; papular eruptions may come out, often accompanied with great itching; bed-sores are common, the motions are passed involuntarily, and saliva dribbles from the mouth. The knee-jerks, which are at first exaggerated, become diminished. The tremors may become so excessive as to shake the bed on which the patient rests; choreiform movements, or even epileptiform seizures are sometimes seen, and rigidity of the muscles of the neck with flexure contractions of the legs on the thighs, and of the thighs on the abdomen, renders the patient's condition a miserable one. The drowsiness passes into coma, from which eventually the patient cannot be roused, the temperature falls to sub-normal, the body and extremities become cold and clammy, and the patient dies. Convulsions in rare instances usher in the fatal termination. This is the usual course of an acute case of the disease, the different changes taking about a month or six weeks for completion. In chronic cases and those treated with atoxyl the symptoms develop more slowly, periods of deceptive improvement, or even apparent cessation of the disease, alternating with periods of advance till at last the final symptoms appear and carry the patient off.

Clinically in the different systems there are special points to note. The temperature is marked in typical cases by an evening rise up to 100 to 104°, followed by a morning fall even of 4°, or more. This regular pyrexia may go on during the whole stage of the disease, or irregularity may set in, or sometimes a normal temperature with slight evening rises. A week or so before death a fall to sub-normal is very common and is a certain sign of a fatal termination. The pulse-rate is 90 to 130 per minute; it often varies considerably during the day, and bears no constant ratio to the temperature, a high frequency being frequently associated with a low temperature. This peculiarity of the pulse is very important in the early diagnosis of human trypanosomiasis. There are no respiratory symptoms, with the exception of the increased rate of 20 to 30. The alimentary, urinary and sexual systems present nothing specially worthy of note. In uncared-for native cases a roughness of the skin with papulo-pustular eruptions is often present, but many retain a perfect smoothness of the epidermis to the end. The circular erythematous rings seen on Europeans suffering from trypanosomiasis are not visible on the black skins of natives. In the lymphatic system a general enlargement of the lymphatic glands is a constant feature in sleeping sickness. The superficial chains can usually be felt in the anterior and posterior triangles of the neck, in the sub-mental and sub-maxillary regions, in Scarpa's triangle and in the groins. Their size varies from a small bean to a hazel-nut, or even larger; they are somewhat hard and firm in consistence, and do not become adherent to, or cause ulceration of, the adjacent skin. Suppurative foci, especially in those about the regions of the mouth, may be encountered. Trypanosomes can readily be demonstrated in the juice withdrawn from these glands by a hypodermic syringe, and this is, perhaps, the best method for diagnosing their presence in the system.

### Blood Changes.

In the hæmopoietic system anæmia in varying degrees is constant, the average number of red cells per cubic millimetre being about 3,000,000. Just before death, in some instances, there may be an abnormal rise to over 6,000,000 per cubic millimetre, but more usually a gradual fall to 2,000,000 or under is observed. The hæmoglobin is generally reduced proportionally to the reds, the blood showing all the characteristics of a well-marked secondary anæmia. The leucocytes are normal or slightly diminished in number; certain cases show a well-marked terminal polymorphonuclear leucocytosis just before death. Differentially the large mononuclear leucocytes are relatively increased. Trypanosomes are present in the blood, but may be very scanty, prolonged search being required to demonstrate them.

The most marked signs of the disease, as the name sleeping sickness suggests, are to be found in the nervous system. The dull apathetic look is characteristic. The expression is heavy and shows very little emotion. There seems to be a slight loss of intelligence, but memory is fairly good. When spoken to, a considerable interval generally elapses before the reply to the question is given. Speech is not specially affected, there being no definite stammering nor slurring in the production of individual syllables. Sleep is in many cases not such a predominant factor as is generally supposed, and it is questionable if the amount is really in excess of the normal. Often when a patient is sitting apparently asleep with his eyes closed, the slightest touch or any trivial noise will cause him to open his eyes at once. Though the total amount of sleep may in some cases be above the average the condition is better defined as one of lethargy, indifference and drowsiness, and even those features in some cases are scarcely noticeable, or may be absent. The motor functions are considerably deranged. The muscles are wasted and flabby, and motor-power diminishes towards the end. In many cases a certain degree of inco-ordination is distinct, and in a few instances Romberg's sign is present. Walfrideson has described a case in which a staggering gait, with a tendency to fall forward, was a prominent symptom, while at the same time there was well-marked lateral nystagmus on looking to the left. Diffuse choroiditis has been noted in a European case of human trypanosomiasis; on the whole, however, eye lesions must be considered as very rare.

### Pathology and Pathological Anatomy.

The lesions found in the organs of people who have succumbed to sleeping sickness are characteristic. Macroscopically there is not very much to note, but microscopically the changes are those of a chronic meningo-encephalitis and meningo-myelitis. Throughout the whole central nervous system, but especially at the base of the brain over the medulla, pons, and cerebellum, the pia-arachnoid is infiltrated with mononuclear cells, and a similar change spreads along the septa and affects in varying degree the perivascular spaces round the blood-vessels. The degree of this perivascular infiltration probably corresponds with that of the lethargic symptoms seen during life. A similar change may also be traced round the vessels in the other organs of the body, especially the heart. The enlarged lymphatic glands and other lymphoid structures show a proliferation of lymphocytes with acute congestive changes. Consequent on these changes, the nerve-cells degenerate and other minuter changes may be detected. The cerebro-spinal fluid is in excess and contains trypanosomes.

### Diagnosis and Prognosis.

All irregular fevers occurring in people who have been living in areas where sleeping sickness is endemic, especially if not influenced by quinine, should be viewed with suspicion. The pulse-rate and respirations should be carefully studied, the skin examined for the circinate erythematous patches, the glands carefully palpated, and the trypanosomes searched for either in the blood, the cerebro-spinal fluid, or the gland juice. They should always be demonstrated before giving a diagnosis and commencing treatment. Since the adoption of the atoxyl treatment there is certainly more hope for early cases of trypanosomiasis, but it is very doubtful if recovery can ever occur after undeniable symptoms of sleeping sickness have appeared.

### Treatment.

Of the many drugs tried in trypanosomiasis of man and animals, none have given such good results as this atoxyl. To Thomas and Breinl, of Liverpool, belongs the credit of first having used it in such cases. Todd recommends that it be given subcutaneously in the form of a 20 per-cent solution in sterile normal saline, the solution being warmed to blood-heat just before use. Of this 0.6 c.cm. is given daily for four days, then 0.8 c.cm. on the four succeeding days, then 1 c.cm. daily for a week; after that 1 c.cm. twice a week until all symptoms have disappeared and the patient's blood is negative to sub-inoculation into susceptible animals. Then 1 c.cm. should be given weekly for as long a period as possible. If signs of poisoning appear the doses must be reduced. More recently Nierenstein and others in Liverpool have advocated the use of mercury and atoxyl, believing that there are two phases or stages of the trypanosomes, the former drug killing the one stage, the latter the other. In addition to the drug treatments the patient's health should be built up by good food, fresh air, and by every other possible means.

